# A Flexible Mechanism of Rule Selection Enables Rapid Feature-Based Reinforcement Learning

**DOI:** 10.3389/fnins.2016.00125

**Published:** 2016-03-30

**Authors:** Matthew Balcarras, Thilo Womelsdorf

**Affiliations:** Department of Biology, Centre for Vision Research, York UniversityToronto, ON, Canada

**Keywords:** value-based decision making, reinforcement learning, rule selection, model-free, cognitive flexibility

## Abstract

Learning in a new environment is influenced by prior learning and experience. Correctly applying a rule that maps a context to stimuli, actions, and outcomes enables faster learning and better outcomes compared to relying on strategies for learning that are ignorant of task structure. However, it is often difficult to know when and how to apply learned rules in new contexts. In our study we explored how subjects employ different strategies for learning the relationship between stimulus features and positive outcomes in a probabilistic task context. We test the hypothesis that task naive subjects will show enhanced learning of feature specific reward associations by switching to the use of an abstract rule that associates stimuli by feature type and restricts selections to that dimension. To test this hypothesis we designed a decision making task where subjects receive probabilistic feedback following choices between pairs of stimuli. In the task, trials are grouped in two contexts by blocks, where in one type of block there is no unique relationship between a specific feature dimension (stimulus shape or color) and positive outcomes, and following an un-cued transition, alternating blocks have outcomes that are linked to either stimulus shape or color. Two-thirds of subjects (*n* = 22/32) exhibited behavior that was best fit by a hierarchical feature-rule model. Supporting the prediction of the model mechanism these subjects showed significantly enhanced performance in feature-reward blocks, and rapidly switched their choice strategy to using abstract feature rules when reward contingencies changed. Choice behavior of other subjects (*n* = 10/32) was fit by a range of alternative reinforcement learning models representing strategies that do not benefit from applying previously learned rules. In summary, these results show that untrained subjects are capable of flexibly shifting between behavioral rules by leveraging simple model-free reinforcement learning and context-specific selections to drive responses.

## Introduction

Successful behavior in new environments benefits from leveraging learning from previous experience in the form of abstract rules—the mapping of contexts, stimuli, actions and outcomes—even though it is often difficult to know which rule is relevant to the current context (Miller, 2000; Gershman et al., 2010a; Buschman et al., 2012; Chumbley et al., 2012; Collins and Frank, 2013; Collins et al., 2014). One of the hallmarks of human behavior is that in new environments with unknown relationships between stimuli and outcomes, subjects generalize from previous experiences (Seger and Peterson, 2013; Collins et al., 2014), even when expectations about the value of stimuli for predicting reward may not be beneficial (Anderson and Yantis, 2013; Shteingart and Loewenstein, 2014). Fortunately, there is significant continuity across our every-day decision making contexts that enables positive transfer of previously learned rules, and in fact, humans work very hard to pattern our living and working environments in such a way as to provide continuity with contextual cues indicating the relevant rule to apply (Gershman et al., 2010b; Collins et al., 2014). For example, objects colored bright red often indicate emergency response equipment, and materials and objects with specific shapes, like octogons, indicate specific information about appropriate responses, like stopping your vehicle. However, people do not always apply rules when it is beneficial to do so. This could be because it is unclear which rule to apply or that an appropriate rule for this context has not been learned.

In this study we set out to test if naive and uncued subjects will spontaneously apply a flexible rule for learning stimulus-feature reward associations and how this behavior can be captured in formal reinforcement learning frameworks. In particular, we explored how subjects leverage an abstract rule that maps stimulus color and shape, independently of each other, to choice outcomes in order to improve the local learning of associations between stimuli and feedback. We hypothesized that untrained subjects exploit previous learning by spontaneously assuming that the feature dimensions of shape and color would be relevant for solving the task and that this would translate into improved performance through a contextually structured selection process.

It is not clear how to formalize the flexible application of behavioral rules in the reinforcement learning (RL) model framework. One solution is to adapt hierarchical RL methods (Collins and Frank, 2013). There is considerable similarity between applying pre-learned rules and hierarchical learning strategies. Structuring stimulus selection hierarchically incorporates expectations about the relevance of stimuli in terms of initiation conditions, the conditions under which an alternate selection sequence is triggered (Botvinick et al., 2009; Badre and Frank, 2012; Botvinick, 2012). Previous work on hierarchical RL has focused on the benefits of temporally abstract actions, where instead of selecting from among available primitive actions; the model can select a behavioral subroutine that employs a sequence of actions. Extending this approach, we developed a model that hierarchically structures the stimulus selection process among competing values for stimulus features. In the default scenario, basic model-free RL learns the expected value of features of visual stimuli and stochastically selects among the values of available stimulus features to receive outcomes (Donoso et al., 2014). Following the hypothesis that subjects have learned from pre-task experience that the feature categories of shape and color are often relevant for local learning, the model compares the total expected value for stimulus features of each type, and when the difference between these total group values crosses a threshold an alternate selection process begins and stimulus selection acts only on the learned value of the relevant subset of features, i.e., the feature type (shape or color) that is greatest (see Materials and Methods). The threshold is an independent model parameter fit to each subject that reflects the confidence of the model in determining a feature-value context. We believe that this adaptation of hierarchical RL represents a simple and intuitive framework for capturing the natural learning processes of untrained subjects in an operant learning environment, and provides testable implications for future research into the neural underpinnings of these processes.

## Materials and methods

### Task design

All experimental procedures were approved by York University's Ethics Review Board. Thirty seven participants from the York University community participated in the experiment (age range 19-35, 26/11 male/female), and all gave their informed consent. Participants were offered an incentive for participating in the form of a gift card valued at $10 CAD. Participants performed the experiment on a touch sensitive Sony Vaio laptop running Windows 8, and Matlab (The Mathworks Inc.) with the Psychophysics toolbox (www.mathworks.com; www.psychtoolbox.org) and custom written Matlab scripts controlling the experiment. The laptop had a 15″ capacitive touch sensitive monitor with a resolution of 1920 × 1080 pixels and a refresh rate of 60 Hz. Stimuli were placed at 4.6 degrees from the central fixation point. The laptop was positioned comfortably, ~50–70 cm, in front of subjects to ease their holding and touching responses. The median temporal delay of the touchscreen responses were in the order of 997 ms (± 26 ms SEM). At the start of the experiment, participants were instructed to use the index finger of their dominant hand to touch one of the two presented stimuli, then use the same finger to hold the spacebar to receive feedback, and to make choices that maximized the number of positive feedbacks.

**Figure 1:**
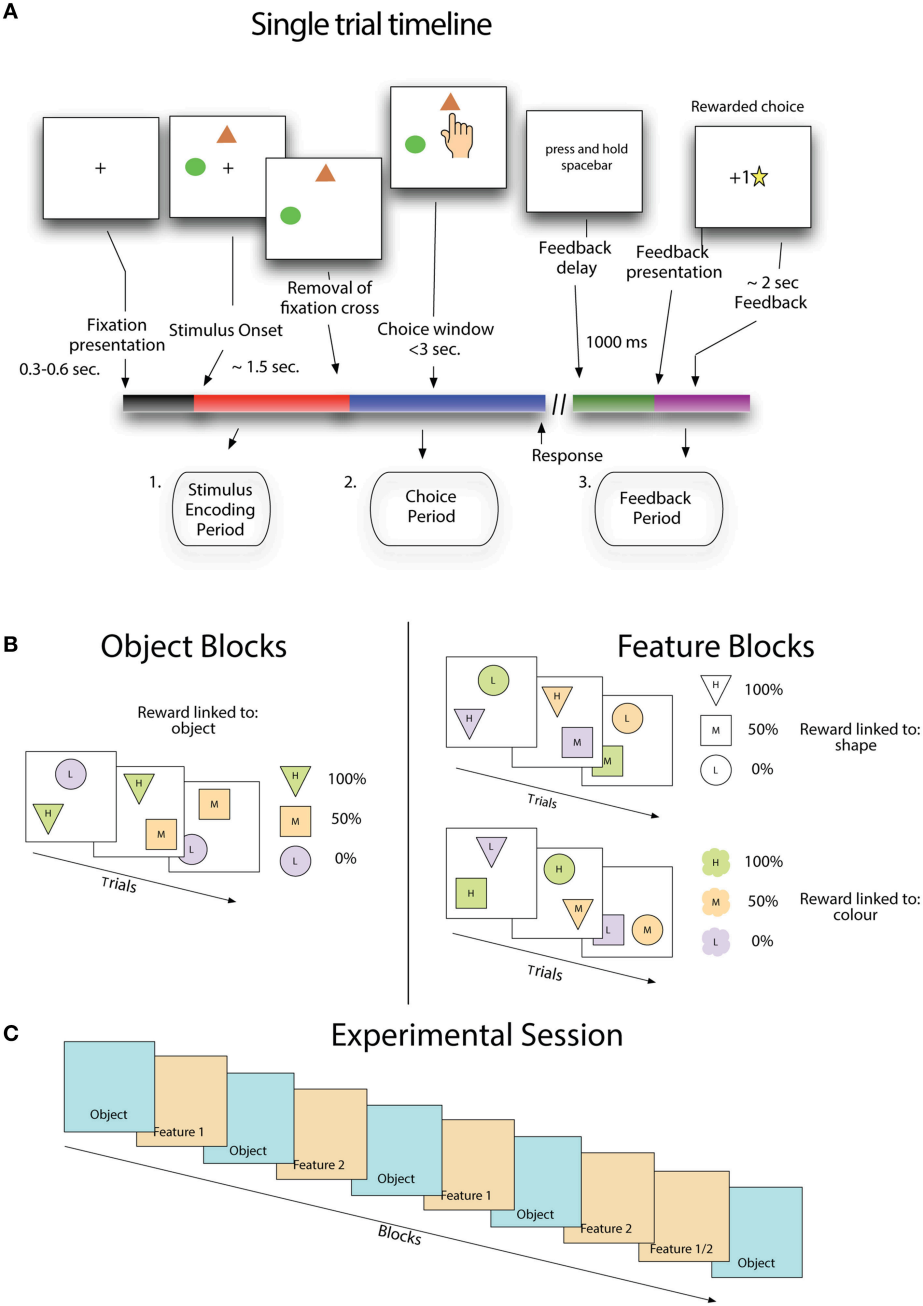
**Stimulus value learning task. (A)** Subjects learned by trial and error that stimuli and stimulus features are linked to the likelihood of receiving positive outcomes. **(B)** Stimulus reward associations were structured either such that fixed pairs of colors and shapes (in sets of three) had a probabilistic relationship with reward (object blocks) or such that stimulus features were not fixed to each other and only one feature type (either shape or color) was linked to reward. **(C)** For the first eight blocks, feature blocks followed object blocks using the same set of shapes and colors as the preceding object block, but with new feature-reward associations. The last pair of blocks flipped this pattern where a feature reward block (either type 1 or 2, randomly selected) precedes an object block.

A trial began with the presentation of a small cross in the center of the screen (Figure 1A). After 300–600 ms two stimuli appeared in two of three possible positions. The location of stimuli was randomly chosen from canonical locations equidistant from each other and the central cross. After another 1500 ms the central cross was removed and subjects were free to select a stimulus. If subjects selected a target before the removal of the fixation cross, the stimuli were removed and a message was displayed reminding the subject to wait for the removal of the cross. This message was displayed for a waiting period of 500 ms before a new trial began. Following the selection of a stimulus, the stimuli were removed and a message appeared on the screen informing subjects to hold the spacebar in order to receive feedback. Feedback was not given until the spacebar was depressed for 1000 ms, and was either a gold star in the middle of the screen or a message saying “sorry” when the schedule associated with that stimulus determined it was either a rewarded or an unrewarded trial (see below). Gold stars awarded to the subject accumulated at the bottom of the screen, indicating to the subjects their performance thus far. After the last trial of the session was completed, a screen was displayed which thanked the subject for participation and provided a final count of gold stars received.

Subjects made choices on stimuli that were combinations of shapes and colors. Each object block began with a new set of three shapes and three colors drawn from a set of six, and all stimuli for that block were made from combinations of these three shapes and colors (Figure 1B). In object blocks, shape-color pairs remain fixed throughout the block so that there is only the unique stimuli appearing in the block. Feature blocks that followed object blocks used the same set of shapes and colors that appeared in the previous block, but now stimuli could be composed of any combination of color and shape, so that there were nine possible unique stimuli appearing in the block.

The task included a hidden probabilistic reward schedule that assigned a probability of positive outcome on each trial to the two available stimuli (Figure 1B - right panels). In object blocks each stimulus, a unique color-shape pair, is assigned a probability of positive outcome, with one being 0%, one 50% and one 100%. In feature blocks, outcome probabilities are associated with a specific feature dimension, either shape or color. In a color-feature block, one color is predictive of positive outcomes 0%, one 50%, and one 100%. Shape-feature blocks work the same as color feature blocks except that probabilities are linked to stimulus shape instead of color. In feature blocks, the non-relevant feature is only spuriously related to outcomes because of the randomized relationship between colors and shapes in these blocks. Receiving a positive outcome for a choice on color A and shape B in a shape-feature block will not tell you anything about the likelihood of receiving a positive outcome on the next trial where color A appears. In both feature and object blocks, stimulus location was selected randomly and was never predictive of outcomes.

Subjects performed a stereotypical sequence of blocks (Figure 1C). An experimental session began with an object block, followed by a feature block, where the relevant feature was selected at random, then another object block and feature block, where this feature is the alternate one from the first feature block. This sequence continued until the ninth block which reversed the object-feature order, and the relevant feature was randomly selected, with the final block being an object block.

Blocks ranged in length from 15 to 25 trials with the number of trials in a block determined by a performance criterion. If the subject had made 11 out of the first 15 choices correctly then the block ended at trial 15. Trials continued until either 80% of the last 10 trials were correct or the subject reached 25 trials. Average block length across subjects was 17.8 (SE ± 2.1). In total subjects performed 7106 trials, of which 3964 trials that were from blocks showing learning were included in analysis.

### Behavioral data analysis

Data Analysis was done with custom written Matlab scripts (The Mathworks Inc.,). Learning in a block was determined following the method of Wilson and Niv (2011), whereby if the slope of the average performance line from the beginning to the end of the block was positive and was above chance performance (50% correct) at the end, the block was considered to show learning.

Correct choices were determined by the selection of the stimulus with the higher probability of a positive outcome, independently of whether a positive outcome was received. On trials where the 100% likely stimulus appeared, it was always the correct stimulus to select, even if selecting the 50% likely stimulus produced a positive outcome. Likewise on trials where the 0% likely stimulus appeared, it was always the incorrect stimulus to select, even if selecting the 50% likely stimulus did not produce a positive outcome.

Reaction times were quantified from the time when the fixation cross was removed until the time when the screen was touched. If the subject touched the screen before the fixation cross was removed the trial was declared an “early response” and was not included in further analysis.

The generalized linear model regression was performed by using (1) the block type or (2) the block number (from 1 to 10), against the mean proportion of correct choices in the whole block of trials, including those blocks that did not show learning overall, in order to determine if block types or repeated exposure to the task over time was predictive of performance. This regression produced a coefficient with a corresponding *p*-value indicating whether the beta-coefficient has a significant predictive relationship with the average performance.

### RL model algorithms

In the basic Q-Learning Rescorla Wagner RL model (QL Basic), the value of any predictor of reward (stimulus feature, Q_i_) is updated on the next time step (trial) from its previous value through the scaled reward-prediction error: The difference between the binary reward outcome (R, either 0 or 1) and the predictor itself (Skvortsova et al., 2014). The scaling factor (α) represents the learning rate:

(1—QL Basic) $$\begin{array}{l} {\text{Q}}_{\text{i}}\left(\text{t}+1\right)={\text{Q}}_{\text{i}}\left(\text{t}\right)+\alpha \left[\text{R }\left(\text{t}\right)-{\text{Q}}_{\text{i}}\left(\text{t}\right)\right] \end{array}$$

Other than the QL Basic model, all other models implemented a generalization of outcome information across all Q values. Thus, all stimulus features associated with the selected stimulus updated their value according to Equation (1). Stimulus features associated with the other, non-selected stimulus were updated according to:

(2—QLGen) $$\begin{array}{l} {\text{Q}}_{\text{i}}\left(\text{t}+1\right)={\text{Q}}_{\text{i}}\left(\text{t}\right)+\alpha \left[1-\text{R }\left(\text{t}\right)-{\text{Q}}_{\text{i}}\left(\text{t}\right)\right] \end{array}$$

The second model, QL Gen, extended QL Basic with generalization of outcome information across all Qs for features appearing on that trial and no other changes. In the third model, QL Decay, feature values were updated when they were associated with the selected stimulus features in the same way as QL Basic and QL Gen, but all non-selected features had their associated values decay as a function of time governed by the rate of decay (τ) according to:

(3—QLDecay) $$\begin{array}{l} {\text{Q}}_{\text{i}}\left(\text{t}+1\right)={\text{Q}}_{\text{i}}\left(\text{t}\right)+\alpha {\left[1-\text{R }\left(\text{t}\right)-{\text{Q}}_{\text{i}}\left(\text{t}\right)\right]}^{*}\tau \end{array}$$

The fourth model, QL GainLoss, employed the same framework as QL Gen, but applied a different learning rate to positive and negative outcomes −α_G_ vs. α_L_.

(4—QLGainLoss) $$\begin{array}{l} {\text{Q}}_{\text{i}}\left(\text{t}+1\right)={\text{Q}}_{\text{i}}\left(\text{t}\right)+\alpha_{\text{G}}\left[\text{R }\left(\text{t}\right)-{\text{Q}}_{\text{i}}\left(\text{t}\right)\right] \end{array}$$

(5—QLGainLoss) $$\begin{array}{l} {\text{Q}}_{\text{i}}\left(\text{t}+1\right)={\text{Q}}_{\text{i}}\left(\text{t}\right)+\alpha_{\text{L}}\;\left[1-\text{R }\left(\text{t}\right)-{\text{Q}}_{\text{i}}\left(\text{t}\right)\right] \end{array}$$

We also created a model of the combination of all parameters previously considered, including α_G_ vs. α_L,_τ for rate of decay, and generalization of outcome information across all Q values. This model is referred to as QL Combined and functions according to:

(6—QLCombined) $$\begin{array}{l} {\text{Q}}_{\text{i}}\left(\text{t}+1\right)={\text{Q}}_{\text{i}}\left(\text{t}\right)+\alpha_{\text{G}}{\left[\text{R }\left(\text{t}\right)-{\text{Q}}_{\text{i}}\left(\text{t}\right)\right]}^{*}\tau \end{array}$$

(7—QLCombined) $$\begin{array}{l} {\text{Q}}_{\text{i}}\left(\text{t}+1\right)={\text{Q}}_{\text{i}}\left(\text{t}\right)+\alpha_{\text{L}}{\left[1-\text{R}\left(\text{t}\right)-{\text{Q}}_{\text{i}}\left(\text{t}\right)\right]}^{*}\tau \end{array}$$

Stimulus feature values for all non-HRL models were non-linearly transformed into choice probabilities according to the Boltzmann equation:

(8) $$\begin{array}{l} {\text{P}}_{\text{i}}\left(\text{t}\right)=\text{e}\beta {\text{Q}}_{\text{i}}\left(\text{t}\right)∕\sum \text{e}\beta {\text{Q}}_{\text{j}}\left(\text{t}\right) \end{array}$$

Where β represents the inverse temperature and establishes the strength of the non-linearity.

The *Flexible Rule Selection model* (FR_Sel) employs a selection function that is an adaptation of the standard Boltzman formulation. Rather than all available Qs competing for final selection via participating as possible choice probabilities, FR_Sel compares Q values across features by feature type, calculating the difference between the sum of total values for each type. When the difference between the total value for one feature type relative to the other types moves past a threshold (λ), only that set of values is used to compute choice probabilities according to the equations below:

(9—FRSel) $${\text{P}}_{\text{i}}(\text{t}){=}_{\text{e}}\beta {\text{Q}}_{\text{i}}\text{(t)/}{\sum_{\text{e}}\beta \text{Q}}_{\text{sel}}(\text{t})$$

Where Q_sel_ is the set of Qs such that:

(10—FRSel.FR_Update) $$\begin{array}{l} {\text{Q}}_{\text{sel}}>\sum {\text{Q}}_{\text{others}}+\lambda \end{array}$$

The *Flexible Rule Update model* (FR_Update) uses the standard Boltzman selection function but employs an update function that restricts the updating of Q values. FR_Update compares Q values by feature type in the same was as FR_Sel (Equation 10) but when the difference between the total value for features by type exceeds λ only the most valued feature values are updated, which takes place according to the most successful non-hierarchical model, QL_Combined.

### Model optimization

Models were optimized by performing a grid search across the total parameter space for each free parameter, attempting to minimize the ordinary least square distance between the probability associated with selecting the correct stimulus and the observed likelihood of selecting the correct stimulus (Bergstra and Bengio, 2012; Donoso et al., 2014; Balcarras et al., 2016) Parameter values were constrained between 0 and 1, which is typical based on the assumption that a value of 0 indicates that term has no predictive power, and 1 indicates the term always predicts the outcome. We did not consider a negative value for lambda. On each trial the model was given the choice made by a subject and transformed that into values according the learning rate(s) of that model iteration. Values were converted into choice probabilities according the Boltzman equation and the value of β (Glimcher, 2011). The mean probability associated with the correct choice was calculated for each trial from the block start across all blocks. Values for free parameters were selected that minimized the distance between this mean probability and the mean likelihood of the subject making a correct choice.

To ensure that we fit the models to the most systematic behavior, we bootstrapped 80% of the data from each subject 100 times for each set of parameter values, and calculated the mean ordinary least squares (OLS) score across these 100 iterations. Bootstrapping is a known method of estimating the variance of model performance (Zucchini, 2000). Because the average performance of the subjects differed across blocks separated by type, we calculated the OLS not only pooled across all blocks, but also for feature blocks and object blocks, which produced a final result for each optimized model in three dimensional OLS space. Models were compared by calculating the Euclidean distance between the combined OLS score and the ideal score of zero. To confirm that optimized model results reflect systematic trends in the data and to correct for model complexity we performed a cross-validation of the model predicted data for each parameter set. Data was split in half by random selection and repeated ten times for each parameter set to ensure that results were consistent independent of data sampling. Using the Wilcoxon-Mann-Whitney test, we found that for each parameter set and each model across all subjects, there was no significant difference in score between data groups (*p* > 0.05) compared to the bootstrapped results.

Computing an OLS value between observed and predicted data is not typical for this method of model-driven hypothesis testing, as compared to the method of finding the maximum LLE, however, the two are not incompatible. We also computed the LLE for each model and each subject and compared the maximum LLE for the best fitting parameter set to the OLS. We found that similarly to what others have found (Donoso et al., 2014) using LLE did not find a more preferable model than the Flexible Rule Selection model (**Figure 5**).

We did not use statistical methods for model comparison, such as the Akaike or Bayesian Information Criterion, because (1) other studies have shown that using OLS is equally capable of identifying the best model (Donoso et al., 2014), and (2) we fit the models to subject performance split by block type, which essentially creates two datasets, and information criterion scores are not comparable across datasets (Zucchini, 2000).

## Results

### Behavior

We show that average choice behavior across subjects is best explained by a reinforcement learning model that identifies the current task context and then applies a selection rule that associates stimuli by feature type and restricts stimulus selection to the relevant (i.e., context specific) stimulus feature. In each trial subjects were required to make choices between two stimuli on a touchscreen and use visual feedback to learn the value of stimulus features for predicting positive outcomes (Figure 1A).

**Figure 2:**
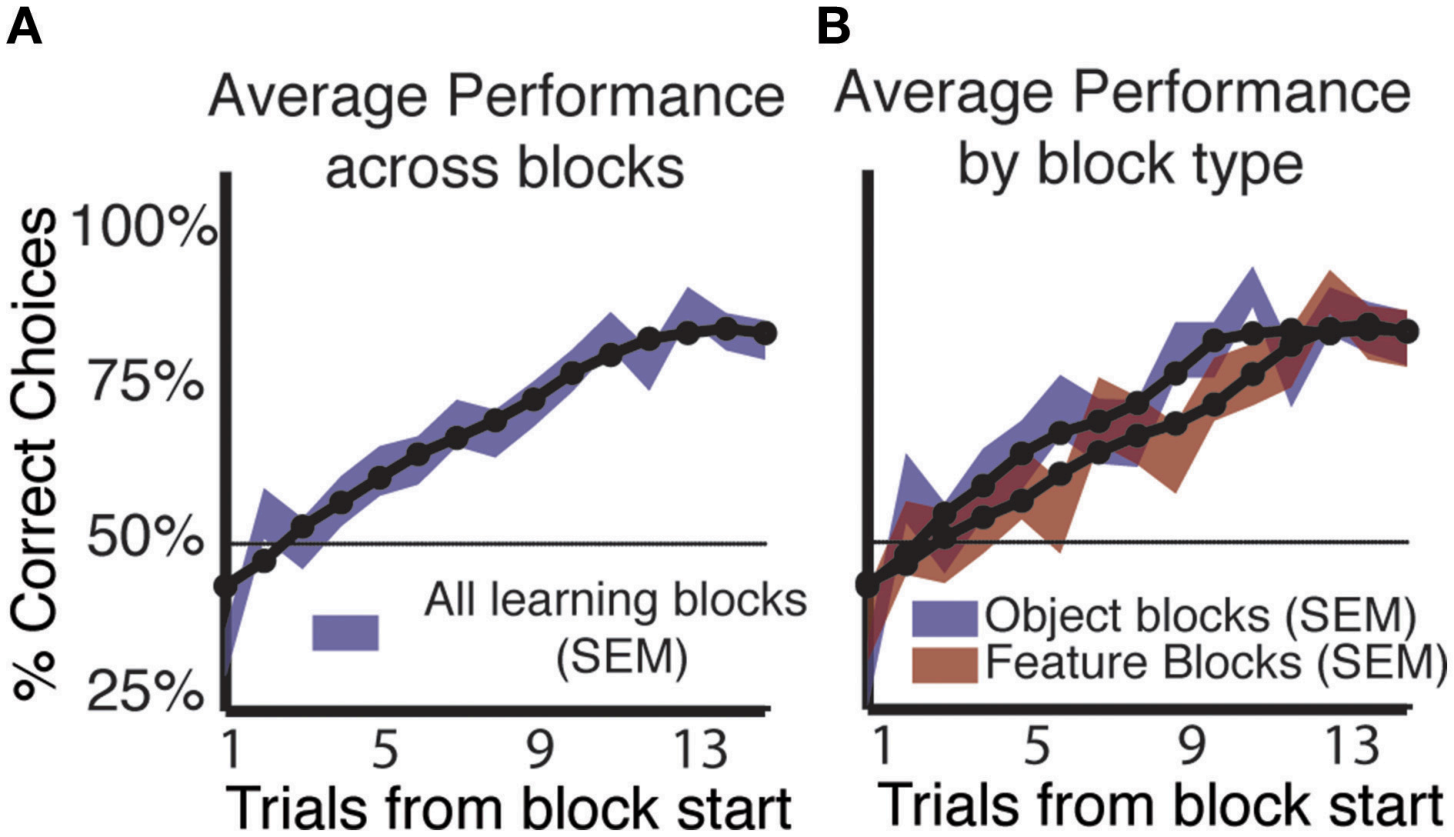
**Learning by block type and across blocks. (A)** Average performance (shading indicates SEM) across all learning blocks shows a consistent increase in the proportion of correct responses for both block types. The 50% line shows chance level performance. **(B)** Splitting average performance by block type shows that performance in object blocks rises faster and peaks sooner than in feature blocks, but that by trial 13 performance in feature blocks equals that of object blocks. Object blocks show significantly better performance compared to feature blocks. (Mann-Whitney-Wilcoxon, *p* < 0.05).

Subjects (*n* = 37, all right handed, 26 male/11 female) were able to successfully use feedback to learn the correct stimulus outcome association in a majority of blocks. Using a simple criterion for learning in a block with constant feature-reward associations (See Materials and Methods, Wilson and Niv, 2011), we found that the majority of subjects (*n* = 32/37) showed learning in on average 158/265 (59.6%) blocks of trials (Figure 2A). Five subjects performed at chance or showed no increase in performance and were excluded from further analysis. Of the 158 blocks in which subjects learned the reward associated rule, 52.5% (83/158) were object blocks. In feature blocks, where only shapes or colors are linked to reward probabilities, subjects showed learning in 47.5% (75/158) of blocks (Figure 2A).

Across subjects and all blocks that showed learning, the proportion of correct choices reached a peak of 82.9% (SE ± 0.03) on trial 15 (Figure 2B). When average performance was split by block type we found that subjects were significantly better in object blocks at trials early in the block compared to feature blocks (Figure 2B). On trials nine and eleven, average performance in object blocks significantly exceeded that in feature blocks by 17.5, and 14.22%, respectively (*p* < 0.05 Mann-Whitney-Wilcoxon). However, at the end of the block, subjects performed equally well in object and feature blocks with identical proportions of 82.89% (SE ± 0.044) −82.86% (SE ± 0.045) correct choices, respectively at trials 12–15 in a block.

To test the hypothesis that performance in a block of trials is related to a learning mechanism that is sensitive to block type, we performed a generalized linear model regression of the proportion of correct responses in a block against the block type sequence, i.e., Object block 1, Feature Block 1, Object 2, etc. We found that this produced a small but significant (*p* = 0.04, β = −0.15) regression coefficient, allowing us to reject the null hypothesis, which indicates that performance is linked to the block type sequence across all subjects. Performing the same GLM by subjects individually found two subjects with a significant coefficient (*p* = 0.0212, β = 0.7843, *p* = 0.0243, β = −0.7738). This suggests that learning in different blocks could be related to a mechanism that responds to the block type. In order to identify this mechanism we developed a range of models discussed below. For comparison, we also regressed the raw block order in a session (Block 1, 2, 3, etc.) against the proportion of correct responses in a block, and we found that this did not result in a significant coefficient (*p* = 0.585 β = −0.002), indicating the performance in a block is not simply a function of time or increased exposure to the task.

### Models

**Figure 3:**
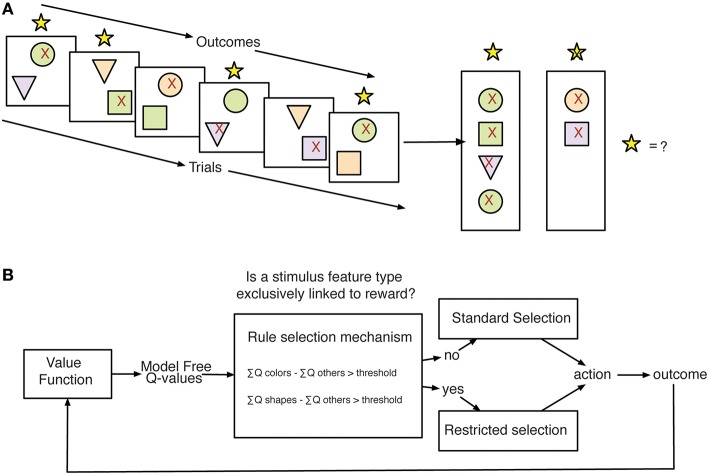
**Stimulus-feature reward association problem and proposed strategy for learning. (A)** Subjects making choices between pairs of stimuli face the problem of learning how to associate stimulus features with outcomes across trials. In the displays, the red 91x92 denotes the chosen stimulus of subjects. The yellow stars on top of each panel indicated the feedback for correctly chosen stimuli. The right panel vertically summarizes the choice out- comes for trials shown on the left to illustrate the subjects putative internal state for determining selections on future trials. **(B)** Model-Schema for Flexible Rule Selection model. The outlined model is proposed as a strategy for solving the learning problem faced in the task. Using learned Q-values for stimulus features, the model compares the sum of values across groups of values separated by feature type. When the difference in the sum of values between feature types grows beyond a threshold the model then restricts selections to the set of Q-values corresponding to the most valuable feature type.

We considered a range of different learning strategies that could be deployed to solve the task through reinforcement learning mechanisms. Each of these strategies was quantified by a separate Q-Learning model (Rescorla, 1976; Cavanagh et al., 2010; Skvortsova et al., 2014) with different functionality representing different assumptions about: (1) the salience of positive vs. negative feedback (QL GainLoss; Materials and Methods, Equations 4 and 5; Gehring and Willoughby, 2002), (2) the impact of time and working memory capacity on learned values (QL Decay; Materials and Methods, Equation 3; Seymour et al., 2012; Skvortsova et al., 2014), (3) the generalization of outcome information across stimulus values (QL Gen; Materials and Methods, Equation 2), and (4) the relevance of subsets of feature values for action selection (Flexible Rule Selection–FR_Sel; Materials and Methods, Equations 7 and 8) (Balcarras et al., 2016). The FR_Sel model was developed in order to capture the hypothesis that subjects would use Q-values for stimulus features to identify a rule for learning, in this case a rule that associates outcomes across trials by feature type and uses the difference in value between types to restrict selection to the most valuable type (Figure 3).

In addition we tested three further models that were combinations of models 1–3.

**Figure 4:**
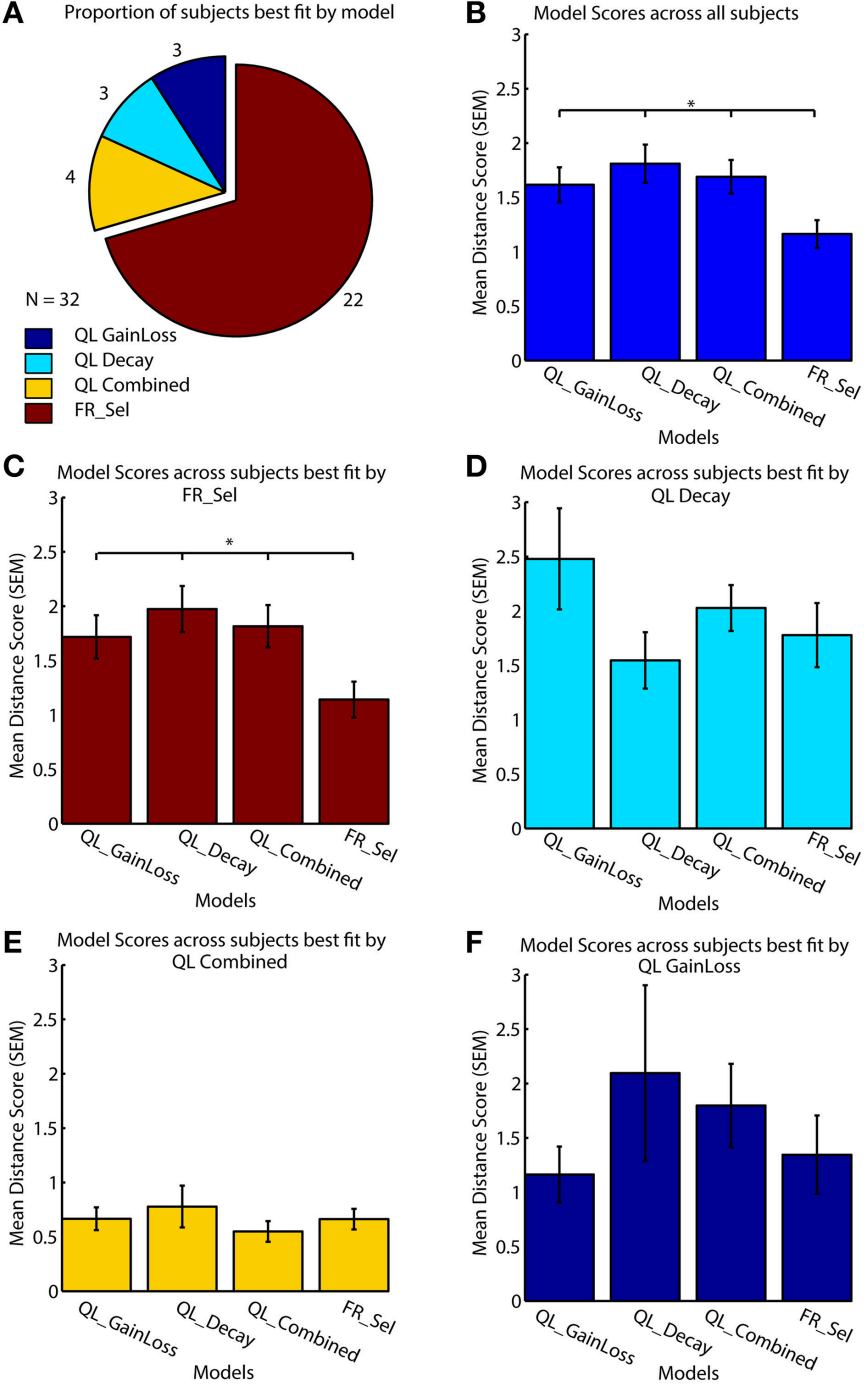
**Model performance across subjects. (A)** All models were fit to each subject individually, with 69% (*n* = 22/32) best fit by the flexible rule selection model (FR_Sel). **(B)** The average ordinary least square score (y-axis) across all subjects for the FR_Sel model was significantly better than all other models for the subjects it fit best. (The star denotes *p* < 0.05, Mann-Whitney-Wilcoxon test, for each pairwise comparison with FR_Sel). **(C)** For subjects best fit by the FR_Sel model, fits with the alternative models were significantly worse. **(D)** For the 11% (*n* = 4) subjects best fit by the QL Decay model, the FR_Sel model provided the second best fit. **(E)** The 11% (*n* = 4) subjects best fit by the QL Combined model showed particular low ordinary least square scores across models. **(F)** For the 5% (*n* = 2) subjects best fit by the QL GainLoss model, the FR_Sel model provided the second best fit.

All models were fit to subject data that showed learning by grid search across the entire parameter space (Table 1, see Materials and Methods; Cavanagh et al., 2010; Donoso et al., 2014; Skvortsova et al., 2014). Four of the seven models considered had at least one subject that was best fit by the model, but the significant majority of subjects (68.75%, 22/32) was fit best by the Flexible Rule Selection (FR_Sel) model (pairwise comparison of bootstrapped OLS scores between all model pairs, Mann-Whitney-Wilcoxon ranksum *p* < 0.05; Figure 4A).

**Table 1:** **Model Names and best fitting parameter values along with measures of fit for individual subjects**.

**Model name**	**Number of parameters**	**Mean (sem) alpha (alphaG)**	**Mean (sem) alphaL**	**Mean (sem) beta**	**Mean (sem) threshold**	**Mean (sem) decay (tau)**	**Mean (sem) OLS distance across all subjects**	**Number of subjects best fit by model**	**Mean (sem) OLS distance across best fit subjects**
**OPTIMIZED MODEL PARAMETERS AND SCORES**									
QL Basic	2	0.552 (0.056)	n/a	0.051 (5e-4)	n/a	n/a	5.5 (0.34)	0	n/a
QL Generalized	2	0.480 (0.052)	n/a	0.053 (5e-4)	n/a	n/a	6.08 (0.394)	0	n/a
QL Decay	3	0.532 (0.054)	n/a	0.061 (5e-4)	n/a	1.04 (0.01)	1.81 (0.175)	3	1.55 (0.26)
QL GainLoss	3	0.457 (0.044)	0.361 (0.045)	0.047 (5e-4)	n/a	n/a	1.62 (0.161)	3	1.16 (0.258)
QL Combined	4	0.373 (0.036)	0.384 (0.055)	0.053 (5e-4)	n/a	1.09 (0.0150	1.69 (0.154)	4	0.545 (0.096)
FR Sel	4	0.373 (0.046)	0.373 (0.052)	0.058 (0.01)	0.244 (0.036)	n/a	1.16 (0.127)	22	1.14 (0.164)
FR Update	4	0.239 (0.023)	0.457 (0.057)	0.037 (0.01)	0.01 (8e-19)	n/a	6.1 (0.398)	0	n/a

*Highlighted row shows scores and parameter values for the Flexible Rule-Selection (FR_Sel) model, which fit 22/32 subjects significantly better than any other subject (Mann-Whitney-Wilcoxon, p < 0.05)*.

The mean score for the FR_Sel model was significantly better than all other models averaged across all subjects (Mann-Whitney-Wilcoxon ranksum *p* < 0.05) and was significantly better than all other models for those subjects that were best fit by the model when tested independently (Mann-Whitney-Wilcoxon ranksum *p* < 0.05; Figures 4B,C). We then quantified how the FR_Sel model scored for subjects whose choices were best fit by one of the other models in order to infer whether these subjects may have used entirely distinct learning strategies, or whether the FR_Sel choice mechanism was still a versatile explanation for these subjects. As shown in Figures 4D–F we found that the FR_Sel model consistently provided the second best explanation for learning choice probabilities in those subjects best fit by the QL Decay model (*n* = 4 subjects, 11%), the QL Combined model (*n* = 4 subjects, 11%), and the QL GainLoss model (*n* = 2 subjects, 5%; Figures 4D–F).

**Figure 5:**
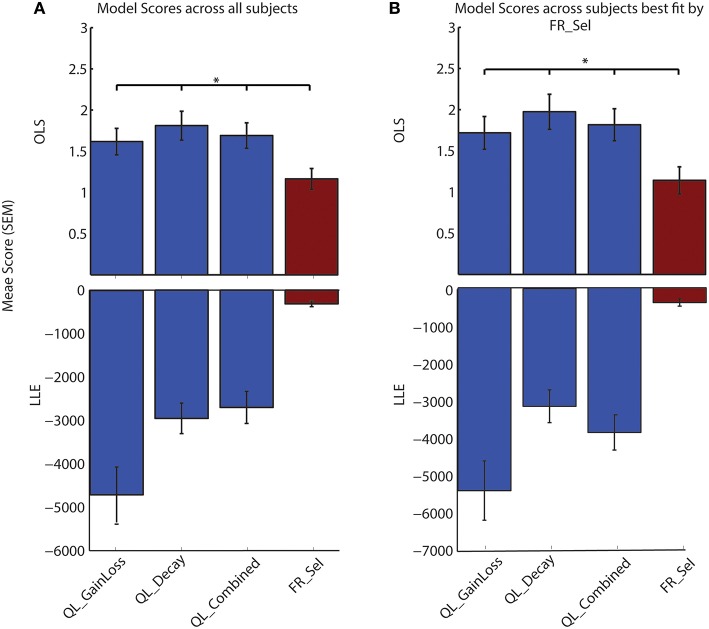
**LLE and OLS methods of model selection both identify FR_Sel as best model**. A comparison of the maximum value for the log-likelihood of model predictions with the ordinary least square value for the difference between observed and predicted choice likelihoods shows that FR_Sel is identified as the best model using both methods. (The star denotes *p* < 0.05, Mann-Whitney-Wilcoxon test, for each pairwise comparison with FR_Sel). **(A)** OLS and LLE scores show FR_Sel as significantly better at predicting choices averaged across all subjects. **(B)** OLS and LLE find FR_Sel significantly better at predicting choices for those subjects best fit by FR_Sel.

A comparison of model selection results using OLS vs. the more typical LLE method confirms that the FR_Sel model is identified as the best model (Figure 5; See Materials and Methods).

**Figure 6:**
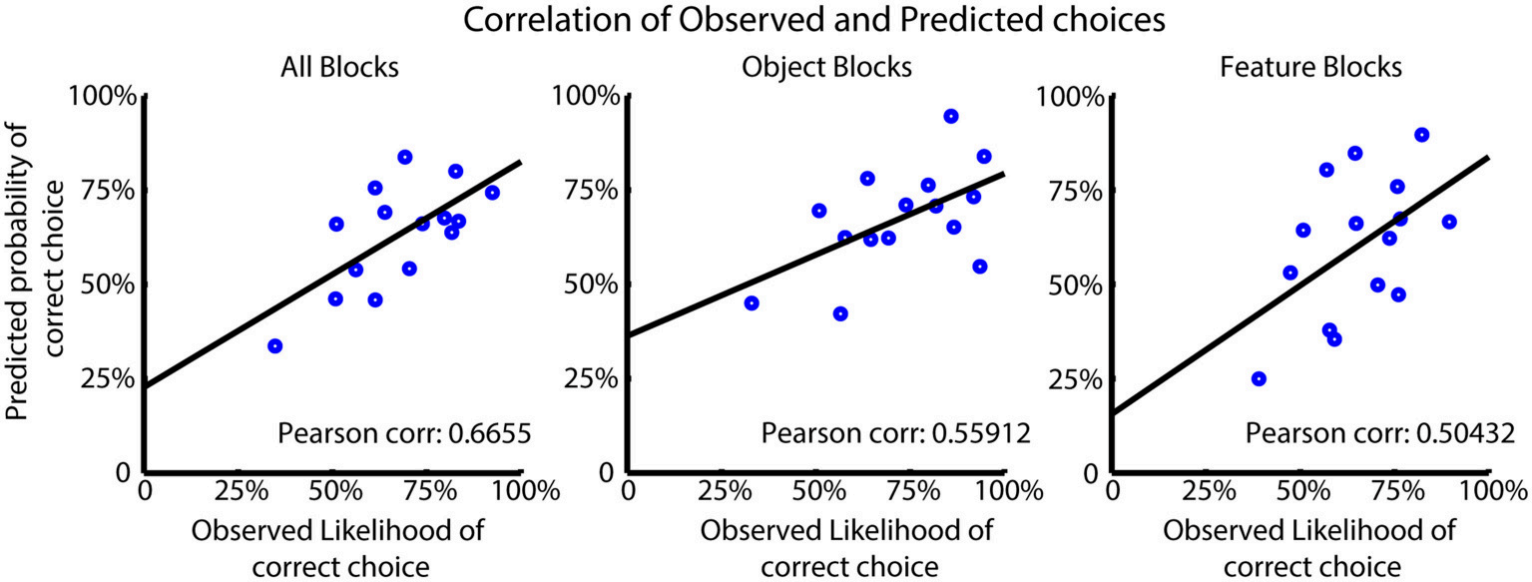
**Model performance for FR_Sel across best fit subjects**. The Pearson correlation was calculated between the mean observed choice likelihood and the predicted probability of making a correct choice based on the FR_Sel model. The significantly correlated (*p* < 0.05 Mann-Whitney-Wilcoxon) predictions produced by the FR_Sel model is shown to illustrate the predictive power of the model for subject behavior.

We tested how the choice probabilities produced by the FR_Sel model predicted the observed likelihood of subjects' correct choices. For this analysis we selected those subjects best fit by the model and computed the Pearson correlation of the average choice probabilities for the model and subjects for all trials, and for trials from feature type and object blocks separately (Figure 6) (see also Balcarras et al., 2016). The FR_Sel model has a significant correlation with the observed data in all block groups (*r* = 0.6655, *p* = 0.001; *r* = 0.56, *p* = 0.012; *r* = 0.50, *p* = 0.01; Pearson correlation) showing that its computed choice probabilities are predictive of average subject behavior.

**Figure 7:**
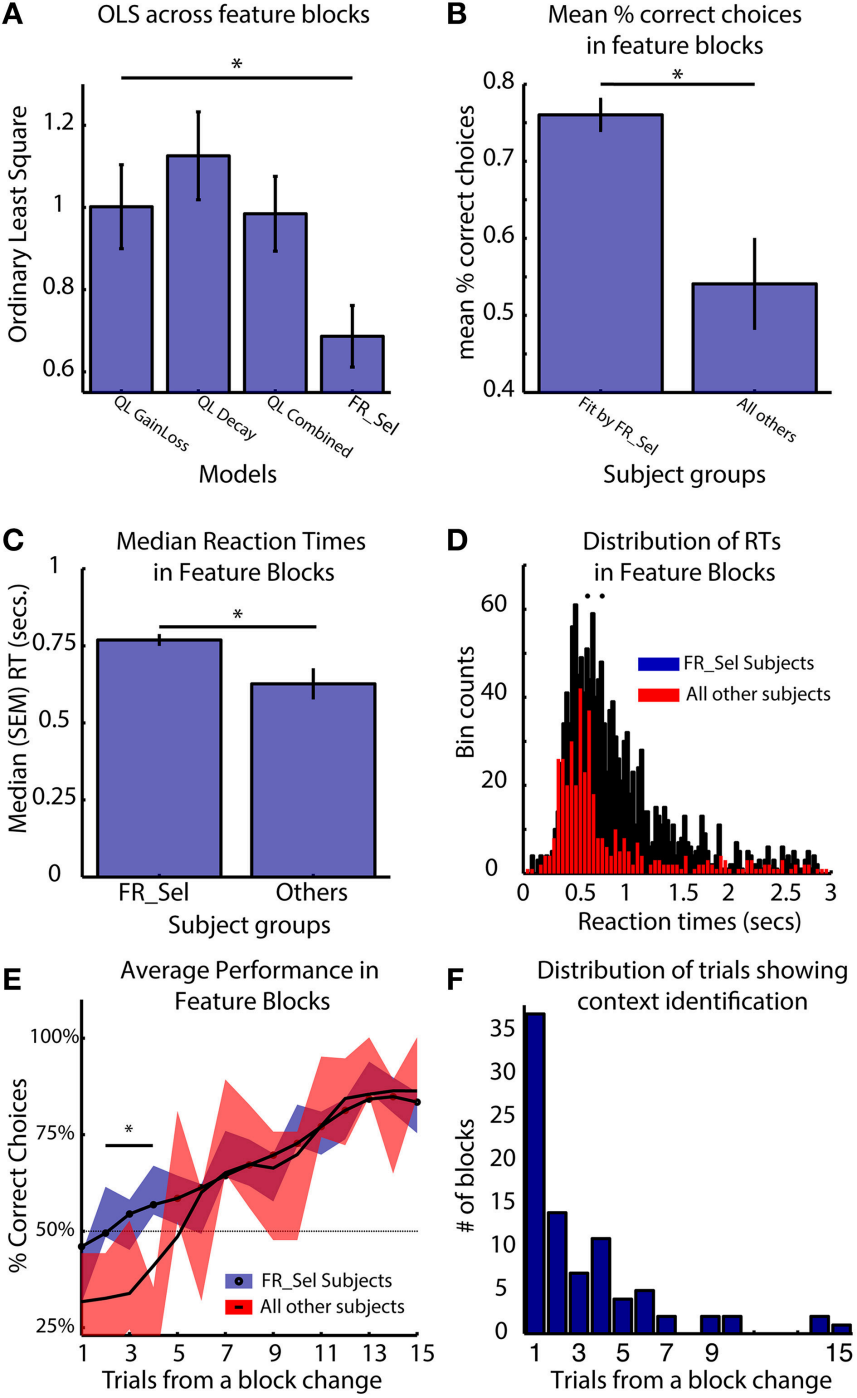
**FR_Sel subjects outperform other subjects, react slower and learn faster**. The star denotes *p* < 0.05, Mann-Whitney-Wilcoxon test for each pairwise comparison. **(A)** For subjects best fit by the FR_Sel model (“FR_Sel subjects”), FR_Sel model predictions in feature blocks was significantly better than all other models. **(B)** FR_Sel subjects (*n* = 22) make on average more correct choices than subjects (*n* = 10) with choice performance that was best fit by other models. **(C,D)** FR_Sel subjects have significantly slower reaction times in feature blocks compared to all other subjects. **(E)** FR_Sel subjects show faster learning in feature blocks, having a significantly higher success rate on early trials in a block compared to other subjects (*p* < 0.05 Mann-Whitney-Wilcoxon). Black line shows smoothed mean of proportion of correct choices, colored traces show unsmoothed mean ± SEM. **(F)** Early responsiveness to the context of feature blocks is predicted by context identification mechanism of the FR_Sel model. The distribution of trials in feature blocks where the model identified the feature specific context is heavily weighted to the first five trials.

The key functional difference between the FR_Sel model and all other models is its ability to flexibly change selection strategies by restricting selection among Q-values to a specific feature domain when the history of choices provided sufficient information about feature type specific gains and losses (see Materials and Methods, Figure 3). Accordingly, subjects using the FR_Sel mechanism (as inferred from being best fit by the FR_Sel model), should show improved performance particularly when transitioning into feature blocks over subjects utilizing other selection strategies (as inferred from being best fit by one of the other models). In support of this suggestion, we found that the FR_Sel model produced the best fit to subject data in feature blocks across all those subjects best fit by FR_Sel according to their overall OLS score (Figure 7A).

Calculating the mean percent correct choices in feature blocks shows that FR_Sel subjects outperformed all other subjects (*p* < 0.05, Mann-Whitney-Wilcoxon; Figure 7B). They also showed significantly slower reaction times (*p* < 0.05, Mann-Whitney-Wilcoxon; Figures 7C,D). Across subjects the median reaction times of subjects did not correlate with the mean % correct choices of subjects (*r* = 0.044, *p* = 0.366). Examining the dynamics of subject performance in feature blocks also showed a significant difference across subject groups. FR_Sel subjects show more rapid learning early in the block, with significantly better performance until trial five (for trial numbers two to five, *p* < 0.05, Mann-Whitney-Wilcoxon; Figure 7E). Faster learning early in the block is another implication of the functionality of the FR_Sel model. Rule deployment specifies context specific selection processes, and this context specific selection, i.e., selection that is restricted to a specific feature domain, is triggered when the difference in values between feature types crossed a threshold. For all subjects best fit by the FR_Sel model this threshold value was quite low (0.21), indicating that very few trials were required to separate values between feature types. With the model generalizing outcome information across chosen and unchosen features, summed values across feature types rapidly diverge. We identified the trials in feature blocks when the FR_Sel model triggered feature specific selection, and plotted the distribution of these trials across blocks (Figure 7F). The model identifies the relevant feature type in the current context rapidly with an average (median) of 2 (SE ± 0.4) trials and with the majority of blocks being identified within the first five trials, which is consistent with the rapid learning early in the block observed in the subject performance.

## Discussion

In this study we tested subjects on their ability to flexibly apply a previously learned abstract rule, respond to uncued context changes, and learn stimulus-feature outcome associations. We developed a set of predictive behavioral models using the reinforcement learning framework, which allowed us to fit the choices of each subject to a unique model, separating subjects that utilize advantageous rule-driven behavior from those that do not. We found that two-thirds of subjects (*n* = 22/32), who were untrained on the task and naive to its design, utilized a strategy for learning that reflected the application of a pre-learned abstract rule relating the association of stimulus feature dimensions to positive outcomes. Importantly, the subjects best fit by the hierarchical rule model were also the subjects that performed the best in more difficult feature blocks, and displayed a significantly slower reaction time on choices in those blocks. Previous studies exploring rule learning and rule driven behavior have focused on either how simple rules are learned via reinforcement, or on how rules can be learned and generalized for application in new contexts. Our study extends this work by quantifying how successful subjects who are naive to the task spontaneously utilize pre-learned task rules to learn in a novel task context.

Rule learning and switching has been studied extensively, typically with a framework such as the Wisconsin Card Sorting Task (WCST) and its analoges (Grant and Berg, 1948; Milner, 1963; Wallis et al., 2001; Buckley et al., 2009; Badre et al., 2010; Mian et al., 2014). In the WCST, four key cards provide the subject with different cues about potential sorting principles across three perceptual dimensions—color, shape and number. Subjects attempt to correctly sort the 128 response cards one at a time, according to the unknown rule, via feedback in the form of binary outcome information (correct vs. incorrect). In our study, we were interested in the flexible application of a more abstract rule, where the rule is informative of a general principle but does not specify the final mapping of a stimulus feature to outcomes, as in the WCST (Bengtsson et al., 2009; Collins and Frank, 2013). Similar to the WCST, the optimal rule to apply in feature blocks is to associate outcomes across trials with a specific feature dimension of the stimulus, however, in our task subjects applying this rule must still learn the specific likelihoods of reward associated with the set of stimulus features presented in that block. For example, after identifying the current context (block) as a color-relevant block, the subject must then learn the rank ordering, or the relative likelihoods of reward, associated with the three colors that appear in that context. Whereas in the WCST, once the stimulus feature sorting rule is learned subjects only need to maintain this rule until it is switched (Stuss et al., 2000; Buckley et al., 2009; Nyhus and Barceló, 2009).

Recently there has been some exploration of how rules are learned and generalized to new contexts. Collins et al. (Collins and Frank, 2013) have shown that subjects are capable of learning rules for task set organization and generalize these rules into new contexts, even when applying a particular rule is not beneficial. Our results are consistent with these findings, with the difference being that those subjects in our study who spontaneously displayed beneficial rule-guided behavior learned the abstract rule prior to the task. Collins et al. use a hierarchical approach to quantify the computational processes associated with abstract rule learning and generalization, similarly to their methods and that of others in the field (Badre and Frank, 2012; Botvinick, 2012; Donoso et al., 2014), we developed a flexible rule selection system that relies on simple model free learning of expected outcomes for stimuli and stimulus features. A model-based approach did not seem appropriate here as rewards were assigned to stimuli stochastically, and transitions between block types were jittered and uncued, all of which prevented subjects from anticipating the likelihood of transitions between states (trials and block types), which is a key functionality of model-based systems.

Many studies of human decision making analyze patterns of choice behavior that collapses subjects into a single unit. This is often done in order to perform analyses of neural activity that averages results across subjects (Cavanagh et al., 2010; Helfinstein et al., 2014; Rudorf and Hare, 2014). While this approach has the benefit of increasing the statistical power of certain techniques it is insensitive to inter-subject variability. Analyzing and fitting models to the systematic behavior of each subject, as we have done here, permits the identification of those patterns of choices that are related to the unique strategy of each subject. It is no surprise to experimentalists that human subjects bring a range of pre-task experiences and expectations to bear on the experimental problem (Shteingart and Loewenstein, 2014), but this is notoriously difficult to account for, and is often just ignored. By using a range of models, each with an associated set of conceptual assumptions, we are able to separate subjects by their flexible application of adaptive rules. While we found that subjects best fit by our flexible rule-selection model were also separable from other subjects according to overall block performance and reactions times, we do not have a hypothesis about why reaction times for these subjects are significantly slower than for other subjects in feature blocks. Further work in this area is needed to clarify the relationship between advantageous use of a flexible rule and reaction times.

Neural activity associated with rule-driven behavior has been found in the prefrontal cortex of humans and non-human primates (Miller, 2000; Bengtsson et al., 2009; Buschman et al., 2012; Womelsdorf and Everling, 2015). Based on the similarity of our results to that of previous studies (Gershman and Niv, 2010; Collins et al., 2014), we would expect to see activity profiles in FR_Sel subjects similar to that found in Collins et al. (2014) and Cavanagh et al. (2010). Because our computational approach produces trial by trial, and subject by subject, estimates of expected values for stimulus features, as well as estimates of trial onsets for rule deployment, simultaneous recording of neural activity in human subjects performing our task would enable sensitive and specific insights into the networks underlying rule deployment and feature value learning. Single trial regression analysis are an underused but powerful tool for investigating the neural mechanisms underlying computational processes implicated in human learning because they compensate for inter-subject variability. Further work on the problem of learning and the ongoing influence of prior learning would likely link lateral PFC areas known to be involved with rule learning and switching to ventromedial PFC areas known to be involved with estimates of stimulus and action values (Wallis et al., 2001; Bengtsson et al., 2009; Buckley et al., 2009; Badre et al., 2010; Gershman et al., 2010a; Wunderlich et al., 2010; Kaping et al., 2011; Mian et al., 2014; Rudorf and Hare, 2014).

## Author contributions

All authors listed, have made substantial, direct, and intellectual contribution to the work, and approved it for publication.

## Funding

Funding for this research was provided by the Canadian Institutes of Health Research (CIHR), the Natural Sciences and Engineering Research Council of Canada (NSERC), and the Ontario Ministry of Economic Development and Innovation (MEDI).

### Conflict of interest statement

The authors declare that the research was conducted in the absence of any commercial or financial relationships that could be construed as a potential conflict of interest. The reviewer JO and the handling Editor declared their shared affiliation, and the handling Edi-tor states that the process nevertheless met the standards of a fair and objective review
